# Clinical Significance of Marital Status and Changes in Status Extracted from Unstructured Clinical Notes Using Ensembles of Off-the-Shelf Extraction Models

**DOI:** 10.21203/rs.3.rs-6578415/v1

**Published:** 2025-05-05

**Authors:** Dmitry A. Scherbakov, Paul M. Heider, Jihad S. Obeid, Alexander V. Alekseyenko, Leslie A. Lenert

**Affiliations:** 1 Medical University of South Carolina, Charleston, South Carolina, USA

**Keywords:** Social determinants of health, marital status, depression, substance abuse, electronic health records, natural language processing

## Abstract

Marital status and its dynamics significantly influence health outcomes. This study aims to demonstrate clinical utility of marital status and improve its extraction from electronic health records (EHR) by developing ensembles capable of using structured and unstructured EHR data.

Four ensembles based on different voting rules (unanimous vote, majority vote, precision-based vote, and vote based on random forest) were assembled using structured marital status data, off-the-shelf CNN, and hybrid models, capable of detecting marital status from clinical notes, followed by BERT-based fine-tuning to increase recall. Associations of marital status and its changes with depression, substance abuse, and other health outcomes derived from MIMIC-III were reported using frequency statistics.

Patients experiencing any marital status change (n=139, including getting married n=40), compared to patients with no change in status (n=2500) of similar age (M=57 years), had higher incidences of depression, but also higher recovery rates, suggesting dual health impacts of marital dynamics.

## Introduction

Marital status and stressful family events, such as divorce, are major predictors of health outcomes^1, 2^. However, accurate marital status data is often missing in electronic health records (EHR) or inconsistently collected during clinical encounters^3, 4^.

Research has highlighted the correlation between marital status and various health outcomes, such as survival rates. Qiu et al. demonstrated that marital status significantly impacts survival rates in osteosarcoma patients, with married individuals showing improved survival compared to single or divorced patients^5^. Pantell et al. found that social isolation, linked to marital status, is a predictor of mortality comparable to traditional clinical risk factors^6^. Similarly, Zhang et al. found that marital status influenced overall survival in patients with soft tissue sarcoma, reinforcing the notion that social support associated with marriage may play a protective role in cancer prognosis^7^.

Hossain et al. highlighted that changes in marital status could significantly affect healthcare service utilization among older adults, indicating that marital dynamics may influence health-seeking behaviors^8^. Furthermore, the literature suggests that marital happiness correlates with better health and longevity, emphasizing the importance of not only marital status but also the quality of the marriage relationship^9^.

In many cases, inferences based on changes in the static structured field “marital status” are the only source of information on family dynamics. Yet, the reliability of the structured marital status field is questionable, and not many EHR systems have convenient access to the history of the changes to static demographic fields. Arguably, analyzing unstructured clinical notes can provide more insight into marital status and its changes. The variability in how marital status is documented across different EHR systems and the potential for ambiguous language in clinical narratives necessitate robust NLP algorithms that can accurately interpret context and meaning^10, 11^. Techniques such as negation detection and temporal relation extraction can further refine the accuracy of NLP applications in this domain, ensuring that the extracted data reflects the true marital status of patients^12^.

The extraction of marital status from clinical records has emerged as a key focus within the broader effort to harness social determinants of health (SDoH) for improving healthcare outcomes. Bucher et al. demonstrated the robustness of rule-based NLP pipelines in determining marital status, achieving an impressive document-level F1 score of 0.97^13^. Their system outperformed a machine learning n-gram model and structured data, showing that unstructured notes provide richer and more accurate insights into marital status. However, their reliance on specific datasets, such as Social Work notes, highlights the importance of data selection in optimizing model performance. Similarly, Lituiev et al. employed hybrid models combining rule-based and machine-learning approaches to extract SDoH information, achieving an F1 score of 80.3%^14^. Feder et al. and Martin et al. explored deep learning techniques, demonstrating that active learning and contextual embeddings (e.g., BERT-based models) significantly improve demographic trait detection in clinical notes, though their work predominantly addressed broader traits beyond marital status^15, 16^. Chen et al. offered valuable insights into the variability and granularity of social history data, indicating that consistency in annotation and extraction frameworks is critical for meaningful integration into clinical workflows^4^.

Across mentioned studies, a recurring theme is the need for iterative model refinement and domain-specific adaptation. While some methods exhibit high precision, they may fail to generalize across institutions due to diverse documentation styles.

In this project, we evaluate several off-the-shelf NLP models for extraction of marital status from clinical notes on the previously unseen MIMIC-III dataset. We hypothesize that their performance can be improved by building an ensemble consisting of off-the-shelf models and a structured EHR “marital status” field. We demonstrate the clinical utility of these improved marital status detection ensembles by using the best-performing ensemble to detect patients whose marital status changed to assess the impact of the change on several health outcomes, including major depression, alcohol, and drug abuse. Thus, this work follows two primary objectives: 1) improve the performance of off-the-shelf models by connecting them into an ensemble; 2) use one of the ensembles to model several health outcomes to demonstrate the clinical significance of marital status and status changes.

## Methods

Our analysis followed these main steps: 1) annotating the data for tuning and testing of the ensembles; 2) building and testing the ensembles alongside base off-the-shelf models and structured “marital status” field; 3) identifying blind spots in the models and structured field data and correcting them by adding a new extraction category (“dating”); 4) building and testing the new, improved ensemble capable of differentiating between “dating” and “partnered” patients; 5) using the improved ensemble to model health outcomes.

### Data for ensemble construction and testing

We extracted social history sections from the MIMIC-III dataset clinical notes, focusing on non-empty entries. A total of 644 sections were manually annotated, forming tuning (n=323) and test (n=321) sets (Table 1).

### Building and testing the ensembles

We used three “off-the-shelf” models: two publicly available NLP models, a CNN model, and a Hybrid model that combines a pattern-matching layer followed by a bag-of-words multinomial logistic regression layer released by Lituiev et al.^14^. These models can predict marital status and living arrangements from clinical notes and the existing MIMIC-III marital status structured field. MIMIC-III structured marital status field was used as an additional source of data when building our ensemble. For CNN and Hybrid models, the “Partnered” status was recoded so that this status is assigned only when the living arrangement “Lives with” label is present in the same note to exclude dating patients who are not cohabitating, at this stage dating individuals are treated as single).

Four ensemble strategies were tested:

1. **Unanimous Voting**: The label is assigned based on the unanimous vote of 3 models
2. **Majority Voting**: The label is assigned based on the majority vote of 3 models
3. **Precision-Based**: The label is assigned based on the model with the highest precision on the tuning set
4. **Random Forest Classifier**: The label is assigned using a random forest classifier trained on the vote of 3 models, and one additional label that is output by CNN and Hybrid models – the living arrangement that captures if the patient is living alone, with family, or others. Thus, the random forest classifier tries to predict the correct label based on 5 labels (3 related to marital status, and 2 to living arrangement).

For ensembles 1 and 2, the label “not given” was assigned in all situations when the label could be set based on the rule. Note that the first two ensembles don’t require a tuning set.

To enhance recall of the resulting ensembles, we utilized a weak supervision approach: four ensemble models generated labels for the remaining portion of the MIMIC-III dataset (n=35358), and the labeled data was used to fine-tune a BERT transformer for each of the four models. All label sources were evaluated on the test split.

### Improving the ensembles by adding a “Dating” category

During the initial testing of the models, it became clear that neither available models nor structured field in MIMIC-III supported the “dating” category. This group is distinct from both partnered individuals who live with their partner, boyfriend, or girlfriend, as well as from “singles” (which typically refers to individuals without a spouse; however, it could be logically extended to exclude individuals who are in a relationship). The structured field tended to view dating individuals as “Single,” while the NLP models treated them as “Partnered.” While during testing, we recoded these individuals by using the “living with” label (from the same models), we wanted to test the possibility of forming a new category instead to have a more granular analysis of the health outcomes. Thus, we improved the detection of dating individuals in NLP models by using an additional fine-tuned BERT model, which we trained on publicly available MIMIC-III annotations related to living arrangements^17^. This model is used to detect whether the patient is living with their partner or not. All patients detected as “partnered” by CNN and the Hybrid model and who don’t live with their partner are coded into a new separate category called “dating.”

After adding a new category, we tested the majority vote ensemble using an additional 650 manually annotated social history sections.

### Evaluating the clinical significance of marital status and changes in status using majority vote ensemble

The improved majority vote ensemble capable of detecting “dating” individuals was then used to bin patients in the whole MIMIC-III dataset into groups of similar age based on their marital status. From this initial sample, we selected patients with at least two intensive care (ICU) admissions no more than 5 years apart. Using the marital status labels from each admission, we further classified patients based on changes in marital status into two groups: “Change in Marital Status” and “Same Marital Status.” We downsampled the larger cohorts so that the mean age in all groups is similar.

For the static marital status patients, we compared occurrences of depression, multiple ICU admissions, alcohol abuse, and substance abuse, along with mean Elixhauser-van Walraven (E-VW) score using derived MIMIC-III data based on International Classification of Diseases (ICD-9) codes^18, 19^. For the dynamic marital status patients, we compared the same health outcomes (except multiple ICU admissions), and additional stress-related health markers calculated from the derived MIMIC-III data: changes in blood pressure, weight changes, and changes in glycated hemoglobin (HbA1C) levels.

## Results

### Testing the ensembles

**Figure 1.**
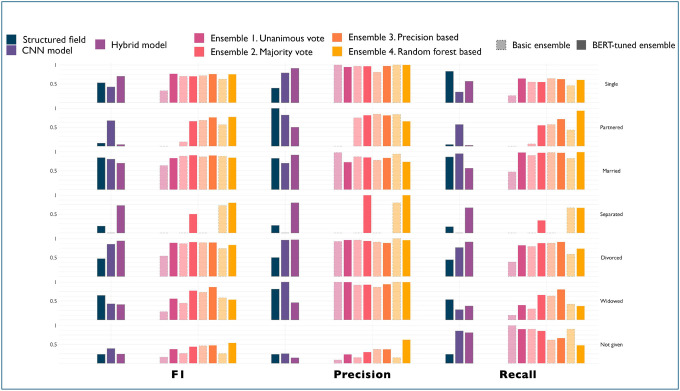
Per class performance of three off-the-shelf models (first three columns), ensemble models (lighter shade and dotted contour), and BERT-tuned ensemble models (darker shade). The ensemble model and paired BERT version are displayed next to each other. Rows contain the marital status labels.

Figure 1 presents the performance of various models for extracting marital status from clinical data, evaluating metrics including F1 score, precision, and recall across different marital status categories. The comparison includes structured data fields, a CNN model, a Hybrid model, and multiple ensemble approaches (unanimous vote, majority vote, precision-based, random forest-based, basic ensemble, and BERT-tuned ensemble). Notably, the BERT-tuned ensembles consistently achieve higher F1 scores across most marital status categories compared to non-BERT versions. Individual models failed to detect some marital status categories: structured field precision was low on single and separated individuals, CNN model precision was low for separated, while Hybrid’s precision was low for partnered and widowed categories.

The simplest form of ensembles based on the unanimous vote and its BERT variant failed to detect partnered and separated individuals. Ensemble methods based on random forests and precision-based aggregation strategies show significant improvements over single models, but only the Random Forest model and its BERT variant were able to detect all marital status labels with relatively high precision.

Table 2 compares the performance of various models and ensemble methods in extracting marital status from clinical data. Metrics include macro- and weighted-average precision, recall, F1 scores, and accuracy across both the tuning (development) and test datasets. Among individual models, the structured field approach exhibits lower overall performance, with macro F1 scores of 0.3733 on the test set. The CNN model performs better with a weighted F1 of 0.6349. The hybrid model achieved a weighted F1 of 0.5566 on the test set.

The random forest ensemble showed high weighted precision on the test set (0.8950), while the BERT-tuned random forest ensemble (Ensemble 4b) showed high macro precision (0.7559) and a weighted F1 (0.6487) on the test set. BERT-tuned precision-based ensemble (Ensemble 3b) scored highest on the weighted F1 score on the test set (0.8070). The majority vote ensemble (Ensemble 2b) came close on weighted F1, achieving a score of 0.7799.

### Improved majority vote ensemble performance

**Figure 2:**
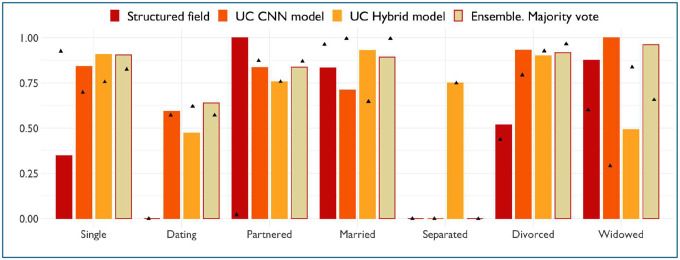
Precision of different models across marital status groups based on 650 notes. Recall displayed as a triangle shape.

The results of testing of improved majority vote ensemble are shown in Figure 2. The ensemble achieved the highest weighted precision – 0.83, while the structured field had the lowest – 0.66 (For our purpose of selecting patients from the dataset for outcomes analysis, higher precision is the most important). Precision and recall per class for each model are shown in Figure 1.

### Comparing health outcomes using improved majority vote ensemble

Table 1 shows the downsampled group sizes for our static and dynamic patient cohorts.

**Figure 3.**
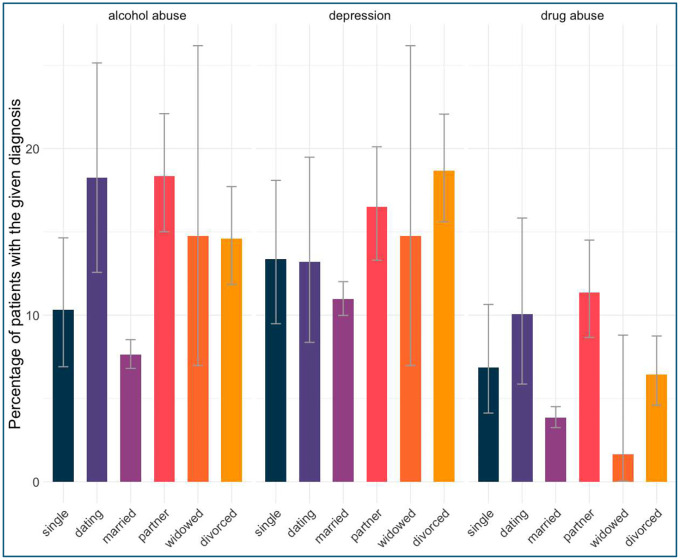
Incidence of depression, alcohol, and drug abuse. All figures are displayed with 95% CI.

As displayed in Figure 3, married patients showed the lowest occurrence of depression (M = 9.32%, 95% CI [8.89%, 9.77%]), drug abuse (M = 2.55%, 95% CI [2.32%, 2.80%]), and alcohol abuse (M = 6.55%, 95% CI [6.18%, 6.93%]). At the same time dating individuals exhibited the highest prevalence of depression (M = 19.3%, 95% CI [15.4%, 23.8%]) and had a high alcohol abuse prevalence (M = 18.3%, 95% CI [14.4%, 22.6%]), while partnered individuals had the highest rates of drug abuse (M = 16.5%, 95% CI [14.4%, 18.8%]) and alcohol abuse (M = 21.8%, 95% CI [19.4%, 24.3%]). Widowed patients had the lowest rates of drug abuse (M = 1.96%, 95% CI [1.20%, 3.02%]) and alcohol abuse (M = 2.36%, 95% CI [1.51%, 3.48%]), but their depression prevalence (M = 12.4%, 95% CI [10.4%, 14.5%]) remained higher than married patients. The mean E-VW score (not displayed) was higher for divorced patients when compared to married patients (Median=7 vs Median= 5, respectively, p < 0.001), but comparison with other groups didn’t reach statistical significance after adjusting for multiple comparisons using the Holm method. Patients who experienced a change in marital status exhibited a higher likelihood of receiving a new diagnosis of depression (M = 7.2%, 95% CI [3.5%, 12.8%]), alcohol abuse (M = 4.3%, 95% CI [1.6%, 9.2%]), and drug abuse (M = 7.2%, 95% CI [3.5%, 12.8%]) compared to those whose marital status remained the same, where the rates of new diagnoses were lower for depression (M = 6.2%, 95% CI [5.2%, 7.2%]), alcohol abuse (M = 1.4%, 95% CI [1.0%, 2.0%]), and drug abuse (M = 1.6%, 95% CI [1.2%, 2.2%]). Similarly, recovery rates for these conditions were also higher among individuals with a marital status change, with 10.8% (95% CI [6.2%, 17.2%]) recovering from depression, 5.0% (95% CI [2.0%, 10.1%]) from alcohol abuse, and 5.8% (95% CI [2.5%, 11.0%]) from drug abuse, compared to those whose marital status remained the same, where recovery rates were lower for depression (M = 4.2%, 95% CI [3.4%, 5.0%]), alcohol abuse (M = 1.8%, 95% CI [1.3%, 2.4%]), and drug abuse (M = 1.6%, 95% CI [1.1%, 2.1%]).

**Figure 4.**
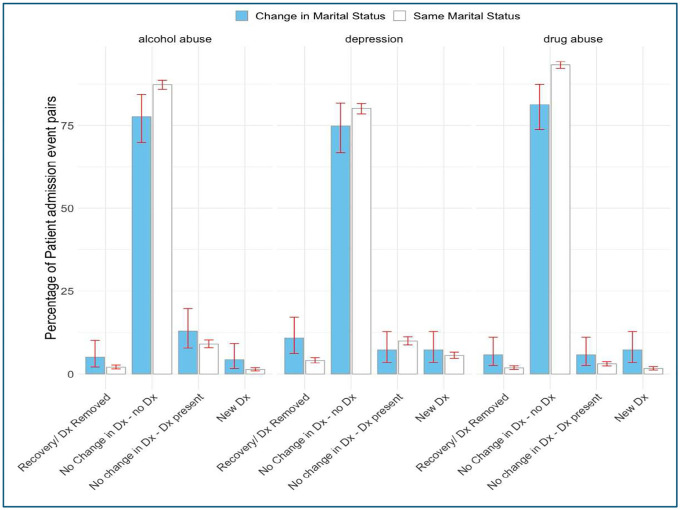
Incidence of depression, alcohol, and drug abuse among patients who had a change in marital status vs. no change group

Similar patterns to Figure 4, although with less statistical significance, were found when assessing separately patients who got married vs. the no change group and when assessing additional health conditions ad-hoc, such as metastatic cancer occurrence and hypertension (not displayed). There was not enough statistical power to detect differences when comparing multiple ICU admissions for static groups and when comparing changes in blood pressure, weight, HbA1C, and E-VW score for “Same Marital Status” vs. “Change in Marital Status” groups. Specific directions of marital status changes and their impact on outcomes were not investigated due to the small sample size in the change group.

## Discussion

### Clinical significance of marital status and changes in status

In agreement with previous studies, we have found that married patients have better health outcomes than other groups. However, when considering changes in status, we found that in the sampled MIMIC-III ICU population, which is older and distinct from the general population, experiencing any change in marital status, including getting married, was associated with higher incidences of depression, drug abuse, and alcohol abuse.

Since changes in status were also linked to higher recovery rates from the abovementioned conditions, we hypothesize that family dynamics may impact health outcomes in two distinct ways. Firstly, marital changes are associated with stress and its detrimental health impact. Loss of a spouse is a source of significant stress^20^, but getting married in older couples is associated with increased demands and pressures, such as health issues in one of the spouses, caring for elderly parents, and financial concerns^21, 22^. Secondly, marriage can increase motivation to seek medical help, and the increased utilization of medical services can lead to both higher diagnosis and recovery rates, the so-called “spousal effect.” This effect involves spouses taking on the role of caregivers, providing both physical and emotional support by promoting healthy behaviors, ensuring partners take their medications as prescribed, recognizing symptoms, and attending medical appointments.

### Clinical utility of ensemble models

This study can serve as a foundational example of how weak supervision and post hoc ensembling can be effectively applied to extract meaningful clinical insights, paving the way for broader applications in healthcare analytics. Simple voting ensembles made from off-the-shelf models are effective mechanisms to boost precision in clinical note extraction when rolling out these models in new environments. At the same time, weak supervision of ensembles using BERT provides an efficient way to enhance recall, which increases the number of patients correctly phenotyped for research or other clinical use cases.

Initial off-the-shelf models showed lower performance on the new dataset than reported by the authors. For example, Lituev et al.^14^ report the F1 score for “single” in the 80s range, while the CNN model didn’t reach 50% and the Hybrid model didn’t reach 75% on our dataset. Similarly, the Hybrid model failed to detect partnered individuals, but in the original study, its performance reached a median of 75%. This underscores the problem of the adaptability of models to new unseen data.

It is important to note that some ensembles require annotated data, such as Precision-based and random forest ensembles, which may be impractical to obtain in a clinical setting. In this case, even the simplest form of ensemble using the majority vote principle is preferred over individual models. This simple form of aggregation produced a remarkable increase in weighted precision, though this model suffered from relatively low recall, which was remediated when performing BERT fine-tuning.

### Limitations

Initial post-hoc significance tests indicate not all numerically different models are significantly different. Additionally, evaluating model performance across different demographic subgroups and EHR systems would be vital to ensure generalizability and equity.

Traditional marital categories used in EHR systems oversimplify the spectrum of relational experiences, even after analyzing available clinical notes data. Incorporating nuanced updates into EHR workflows like “started/ended short-term relationship,” or “long-term relationship started/dissolved”, “dating partner passed away”, etc. could better reflect this diversity and enhance our understanding of health impacts tied to relational transitions^3^.

### Next steps

Our current work focuses on validation of the results published in this paper using the Medical University of South Carolina EHR database covering the population of South Carolina (~approx. two million patients).

## Conclusion

We have demonstrated how to utilize NLP models to extract marital status and status changes from previously unseen clinical notes and how to use the labeled data in clinical analysis. The findings in our study indicate that marriage and other related events can have a significant impact on health. Future research should explore whether changes in marital status affect health outcomes through increased use of medical services, through stressful impact on individuals, or a combination of both. This can be achieved by analyzing additional factors in EHR data, such as an increase in the number of visits to providers after marriage, divorce, and other stressful events. An essential part of future research is taking steps to protect vulnerable groups, such as people in marriage-like relationships, from enforcing stigma related to their health outcomes, but at the same time making sure their health outcomes are recognized and detected by NLP systems, this includes individuals who lose their long or short-term relationship partner (this could be distinct groups from “widowed” and “divorced,” in the same way as we differentiate “partnered,” “dating,” and “married”).

Post-hoc ensembling alone or in combination with BERT-based weak supervision offers robust strategies for improving NLP model performance in clinical phenotyping tasks. Future work could also be focused on extending model performance to encompass patients without a social history section in a discharge summary, using information from other types of notes, such as nursing notes.

## Figures and Tables

**Table 1.** The manually annotated dataset is used for model development.

	Tune set	Test set
**Single** *	53	53
**Partnered**	49	49
**Engaged** **	1	0
**Married**	117	117
**Separated**	6	6
**Divorced**	34	34
**Widowed**	42	41
**Not given**	21	21

Note:

* At this stage, dating individuals are included in the Single group;

** Engaged class not used in the analysis and not displayed in Results (Figure 1) due to an insufficient sample of engaged individuals. However, it was used in calculating performance metrics (Table 2).

**Table 2.** Performance of all models on the tuning and test sets. Metrics are calculated using sci-kit-learn definitions of micro-average (accuracy), macro-average, and weighted averages. Bold font highlights models with the highest precision (our primary target metric) on the tune and test set and the highest F1 on the test set.

	Tune (dev) set							Test set						
		Macro			Weighted			Accuracy	Macro			Weighted		
		Precision	Recall	F1	Precision	Recall	F1		Precision	Recall	F1	Precision	Recall	F1
Structured field	0.5882	0.3348	0.3699	0.3429	0.5284	0.5882	0.5463	0.5919	0.4958	0.3896	0.3733	0.6945	0.5919	0.5583
CNN Model	0.6780	0.5834	0.4926	0.4747	0.7865	0.6780	0.6705	0.6511	0.5660	0.4617	0.4453	0.7568	0.6511	0.6349
Hybrid model	0.5356	0.6165	0.5113	0.4826	0.8190	0.5356	0.5887	0.5109	0.5866	0.4880	0.4690	0.7456	0.5109	0.5566
Ensemble 1. Unanimous vote	0.3437	0.5090	0.2895	0.2655	0.7607	0.3437	0.4134	0.3240	0.5000	0.2704	0.2345	0.7550	0.3240	0.3800
Ensemble 2. Majority vote	0.6471	0.6119	0.4667	0.4362	0.8706	0.6471	0.6593	0.6106	0.5783	0.4382	0.4096	0.8208	0.6106	0.6221
Ensemble 3. Precision based	0.7802	0.5758	0.5573	0.5557	0.7883	0.7802	0.7716	0.7664	0.5728	0.5414	0.5449	0.7777	0.7664	0.7575
Ensemble 4. Random forest based	0.6997	**0.7342**	0.5913	0.6015	**0.8951**	0.6997	0.7507	0.6293	0.7164	0.5356	0.5480	**0.8950**	0.6293	0.6947
Ensemble 1b. BERT+Unanimous vote	0.6594	0.4610	0.4656	0.4149	0.6463	0.6594	0.6083	0.6604	0.4835	0.4679	0.4275	0.6649	0.6604	0.6201
Ensemble 2b. BERT+Majority vote	0.7523	0.6074	0.5525	0.5370	0.8415	0.7523	0.7590	0.7695	0.7249	0.6005	0.6107	0.8508	0.7695	0.7799
Ensemble 3b. BERT+Precision based	0.8297	0.5997	0.6018	0.5915	0.8354	0.8297	0.8232	0.8100	0.6025	0.5868	0.5828	0.8284	0.8100	**0.8070**
Ensemble 4b. BERT+Random Forest	0.7585	0.7233	0.5819	0.6147	0.7941	0.7585	0.7382	0.7788	**0.7559**	0.6139	**0.6487**	0.8244	0.7788	0.7629

**Table 1.** Resulting group sizes after sampling from MIMIC-III corpus. Separated group not used. Mean age ~57 years.

Group	Group size	Group	Group	Group	Group size
Single	262	Married	3774	Same Marital Status	2500
Dating	159	Widowed	62	Change in Marital Status (Got married)	40
Partnered	486	Divorced	592	Change in Marital Status (Other change)	99

## Data Availability

The data underlying this article is available for registered and accredited users in the PhysioNet platform^23^. Our analysis code is available on GitHub: https://github.com/scherbakovdmitri/MIMIC-III-MaritalStatus.
